# Secondary hyperperfusion injury following surgical evacuation for acute isolated epidural hematoma with concurrent cerebral herniation

**DOI:** 10.3389/fneur.2023.1141395

**Published:** 2023-04-17

**Authors:** Wei Huang, Jun Li, Wen-hao Wang, Yuan Zhang, Fei Luo, Lian-Shui Hu, Jun-Ming Lin

**Affiliations:** Department of Neurosurgery, The 909th Hospital, School of Medicine, Xiamen University, Zhangzhou, China

**Keywords:** acute epidural haematoma, brain herniation, cerebrovascular autoregulation, reperfusion injury, secondary brain injury

## Abstract

**Objective:**

Hemispherical cerebral swelling or even encephalocele after head trauma is a common complication and has been well elucidated previously. However, few studies have focused on the secondary brain hemorrhage or edema occurring regionally but not hemispherically in the cerebral parenchyma just underneath the surgically evacuated hematoma during or at a very early stage post-surgery.

**Methods:**

In order to explore the characteristics, hemodynamic mechanisms, and optimized treatment of a novel peri-operative complication in patients with isolated acute epidural hematoma (EDH), clinical data of 157 patients with acute-isolated EDH who underwent surgical intervention were reviewed retrospectively. Risk factors including demographic characteristics, admission Glasgow Coma Score, preoperative hemorrhagic shock, anatomical location, and morphological parameters of epidural hematoma, as well as the extent and duration of cerebral herniation on physical examination and radiographic evaluation were considered.

**Results:**

It suggested that secondary intracerebral hemorrhage or edema was determined in 12 of 157 patients within 6 h after surgical hematoma evacuation. It was featured by remarkable, regional hyperperfusion on the computed tomography (CT) perfusion images and associated with a relatively poor neurological prognosis. In addition to concurrent cerebral herniation, which was found to be a prerequisite for the development of this novel complication, multivariate logistic regression further showed four independent risk factors contributing to this type of secondary hyperperfusion injury: cerebral herniation that lasted longer than 2 h, hematomas that were located in the non-temporal region, hematomas that were thicker than 40 mm, and hematomas occurring in pediatric and elderly patients.

**Conclusion:**

Secondary brain hemorrhage or edema occurring within an early perioperative period of hematoma-evacuation craniotomy for acute-isolated EDH is a rarely described hyperperfusion injury. Because it plays an important prognostic influence on patients’ neurological recovery, optimized treatment should be given to block or reduce the consequent secondary brain injuries.

## Introduction

Isolated acute epidural hematoma (EDH) is one of the most common intracranial injuries, accounting for 2.7–11% of all intracranial injury cases (1). Though timely surgical intervention usually achieves satisfactory outcomes (2), some potential complications can still seriously affect the prognosis (3, 4). Among these complications, intracerebral hemorrhage or edema caused by hyperperfusion during or shortly after surgery causes special attention due to its high mortality and disability (5). However, few studies have reported its characteristics and risk factors. In this study, we retrospectively analyzed patients with isolated EDH who underwent craniotomy in our hospital. Those with intracerebral hemorrhage or edema were identified, and their characteristics were reported in order to have a better understanding of this complication.

## Methods

### Participants

This retrospective study was based on patients admitted into our department between June 2009 and May 2020. The diagnosis was confirmed by the head trauma history, clinical presentations, and radiological findings. The inclusion criteria were as follows: (1) isolated supratentorial EDHs confirmed by computed tomography (CT); (2) patients with cerebral herniation; and (3) patients who underwent craniotomy or decompressive craniectomy. The exclusion criteria were as follows: (1) those with severe heart, lung, and other systemic diseases; (2) those with serious brain contusion and laceration, subdural hematoma, and primary brain stem injury; (3) those with infratentorial EDH or EDH straddling the transverse sinus; (4) those with secondary cerebral infarction; and (5) patients who had incomplete clinical data, including the 3-month follow-up. The research was reviewed and approved by the Institutional Review Board of the 909th Hospital, School of Medicine, Xiamen University.

### Clinical management

A head CT scan was performed for all patients after admission. Those who were suspected of having a risk of secondary brain injury would receive an additional CT angiography (CTA) and CT perfusion (CTP) examination. A craniotomy or a decompressive craniectomy was performed as soon as possible if necessary. Postsurgical management included intracranial pressure (ICP) monitoring, seizure prophylaxis, and nutritional support. Tracheal intubation and mechanical ventilation were adopted based on patients’ consciousness and arterial oxygen saturation.

### Radiographic evaluation

Patients received regular CT examinations before and 1, 3, and 7 days after surgery. CT images were evaluated by two independent senior neuroradiologists with the assistance of clinical neurosurgeons. Diffusion-weighted imaging/apparent diffusion coefficient/magnetic resonance imaging (DWI/ADC-MRI) was applied to confirm if there were *de novo* low-dense lesions in the secondary hyperperfusion.

### Neurological outcomes at follow-ups

Patients were followed at 1 and 3 months after discharge with CT imaging and physical examinations. Thereafter, follow-ups were performed either by telephone interview or by outpatient examination at an interval of 6 months. Neurological outcome was evaluated by using the glasgow outcome scale (GOS) as follows: I, death; II, vegetative survival in a long-term coma; III, severe disability needing care providers; IV, mild disability, being able to take care of daily life; and V, good outcomes, with adults being able to work and study.

### Statistical analysis

Collected data included age, gender, and GCS score upon admission. Data distribution was evaluated by histogram and the Kolmogorov–Smirnov test. Continuous data were either expressed as mean ± standard deviation (SD, statistical analysis: Student’s *t*-test) or median and interquartile range (IQR, statistical analysis: Mann–Whitney *U*-test). Categorical variables were analyzed by the Pearson *χ*^2^ or Fisher exact test. Parameters with a *p*-value of less than 0.2 in the univariate analysis were included in the multivariable logistic regression analysis. All the statistical analyses were performed with SPSS, and a *p*-value of less than 0.05 was considered to be statistically significant.

## Results

In total, 157 patients with isolated supratentorial EDHs who had craniotomy or decompressive craniectomy in our department between June 2009 and May 2020 were included in our study. There were 134 (85.35%) male patients and 23 (14.65%) female patients, with a mean age of 35.20 ± 14.58 years. The injury mechanism included traffic accidents (120/157), assault injuries (19/157), and falling injuries (18/157). The hematoma was located in the temporal area in 89 (56.69%) cases, and in the non-temporal areas, it was seen in 68 (43.31%) cases. Preoperative unilateral pupil dilation was found in 127 (80.89%) cases and bilateral pupil dilation in 30 (19.11%) cases. The mean duration of brain herniation was 73.7 ± 36.6 min, and the mean GCS on admission was 7.30 ± 2.32 points.

Diffusion-weighted/apparent diffusion coefficient magnetic resonance imaging (DWI/ADC-MRI) and CTP examinations were performed to clarify whether the secondary brain hemorrhage or edema was an ischemic lesion or hyperperfusion lesion. Among the 206 patients, 12 cases were found to have secondary hemorrhage or edema, confirmed with hyperperfusion by CTP (Table 1), while another 145 were indicated to have normal cerebral perfusion. The characteristics of patients with a hyperperfusion lesion and normal cerebral perfusion are presented in Table 2. Patients with a hyperperfusion lesion had a lower GCS score, longer duration of preoperative herniation, larger EDH volume and thickness, and were more likely to have non-temporal EDH location compared to those with normal perfusion. In the multivariable logistic regression analysis, the duration of preoperative herniation and EDH thickness were independently associated with the risk of hyperperfusion injury (Table 3).

**Table 1:** CTP parameters of 12 patients with secondary brain hemorrhage or edema due to hyperperfusion.

CTP parameters	hyperperfusion lesion region	Contralateral mirror region	*p*-value
CBF[mL/(100 g.min)]	67.43 ± 7.48	43.98 ± 5.00	<0.001
CBV[mL/100 g]	5.31 ± 0.64	3.82 ± 0.47	<0.001
MTT(s)	2.72 ± 0.38	3.40 ± 0.42	<0.001

CBF, cerebral blood flow; CBV, cerebral blood volume; MTT, mean transit time.

**Table 2:** Clinical characteristics of patients with normal perfusion and hyperperfusion.

Clinical parameters	Risk stratification	Normal perfusion	Hyperperfusion	*P*-value
*n* (%)		145	12	-
Sex	Female	26 (89.66)	3 (10.34)	0.465
	Male	119 (92.97)	9 (7.03)	
Age (year)	-	34.94 ± 14.59	37.67 ± 20.42	0.549
	12–65	138 (93.88)	9 (6.12)	0.030
	≤12 or ≥ 65	7 (70.00)	3 (30.00)	
Shock	No	135 (92.47)	11 (7.53)	0.595
	Yes	10 (90.91)	1 (9.09)	
GCS	-	7.99 ± 2.05	6.33 ± 0.98	<0.001
	≤5	10 (90.91)	1 (9.09)	0.595
	≥6	135 (92.47)	11 (7.53)	
Duration of preoperative herniation (min)	-	60.14 ± 24.87	115.00 ± 44.21	<0.001
	<60	74 (97.37)	2 (2.63)	<0.001
	60–120	66 (94.29)	3 (4.35)	
	≥120	5 (41.67)	7 (58.33)	
Admission pupil state	Unilateral dilation	137 (93.20)	10 (6.80)	0.171
	Bilateral dilation	8 (80.00)	2 (20.00)	
Basal cistern	Narrowed	106 (92.98)	8 (7.02)	0.737
	Disappeared	39 (90.70)	4 (9.30)	
Midline shift (mm)	-	10.58 ± 2.23	11.83 ± 1.19	0.057
	<10	39 (97.50)	1 (2.50)	0.446
	≥10	106 (90.60)	11 (9.40)	
EDH location	Non-temporal	65 (86.67)	10 (13.33)	0.014
	Temporal	80 (97.56)	2 (2.44)	
EDH volume (mL)	-	89.83 ± 29.50	114.08 ± 31.62	0.007
	<120	123 (95.35)	6 (4.65)	0.008
	≥120	22 (78.57)	6 (21.43)	
EDH diameter (cm)	-	9.18 ± 1.28	8.58 ± 1.12	0.113
	<10	103 (90.35)	11 (9.65)	0.229
	≥10	42 (97.67)	1 (2.33)	
EDH thickness (mm)	-	29.70 ± 6.27	39.50 ± 6.20	<0.001
	<35	120 (97.56)	3 (2.44)	<0.001
	35–40	15 (88.24)	2 (11.76)	
	≥40	10 (58.82)	7 (41.18)	

EDH, epidural hematoma; GCS, glasgow coma scale.

**Table 3:** Multivariate logistic regression analysis for the risk of secondary reperfusion injury following surgical evacuation of acute EDH.

Clinical factors	Risk stratification	Partial regression coefficient	Relative risk (95% CI)	*P*-value
Duration of preoperative herniation, min	60–120	1.230	3.422 (0.481–24.349)	0.219
	≥120	4.121	61.617 (5.851–648.890)	0.001
EDH thickness, mm	35–40	1.084	2.956 (0.289–30.239)	0.361
	≥40	2.308	10.051 (1.478–68.366)	0.018
Age, yr	<12或 > 65	1.744	5.723 (0.633–51.770)	0.121
EDH location	Non-temporal	1.706	5.507 (0.734–41.306)	0.097

EDH, epidural hematoma.

Patients with hyperperfusion injury had significantly worse clinical outcomes compared to those with normal perfusion (3-month GOS score, 3.83 vs. 4.89, *p* = 0.011; Table 4). Among the 12 patients with hyperperfusion injury, two had unresponsive wakefulness (GOS 2), three had a severe disability (GOS 3), two had a mild disability (GOS 4), and five had a good recovery (GOS 5) at a 3-month follow-up.

**Table 4:** Clinical outcomes in the included patients (%).

Clinical parameters	Risk stratification	Normal perfusion	Hyperperfusion	*P*-value
*n* (%)	-	145 (70.39)	12 (5.83)	-
3-Month GOS	-	4.89 ± 0.47	3.83 ± 1.19	0.011
	1	0	0	<0.001
	2	0	2 (16.67)	
	3	2 (1.38)	3 (25.00)	
	4	9 (6.21)	2 (16.67)	
	5	134 (92.41)	5 (41.67)	

GOS, glasgow outcome scale.

### Case 1

**Figure 1:**
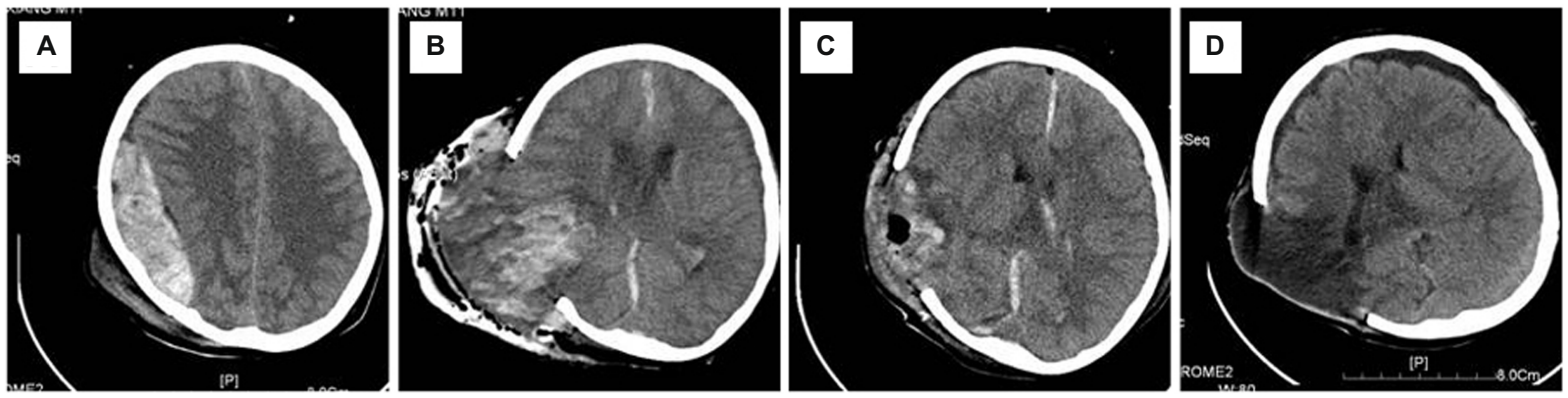
Representative radiographic images of Case 1. **(A)** An acute epidural hematoma in the right temporal–parietal area; **(B)** regional but not hemispherical bulging of the brain tissue occurred after hematoma evacuation, and intra-operative CT confirmed that there was no remote or contralateral hematoma except a “flame”-like intraparenchymal hemorrhage in the hematoma-compressed area; **(C)** incarcerated and necrotic cerebral parenchyma was resected, and the remaining brain tissue became atonic; **(D)** 1-month CT follow-up showed the presence of a focal encephalomalacia in the affected area and a contralateral subdural effusion, without any remote cerebral infarction. CT, computerized tomography.

As shown in Figure 1, a 12-year-old boy was admitted with hemorrhagic shock after a traffic accident (GCS 5). Physical examination showed right pupil dilation (5 mm) and left pupil fixation (3 mm). Emergency CT depicted an acute epidural hematoma in the right temporal-parietal area with a volume of 83 ml and a thickness of 31 mm. After a preoperative emergency burr hole draining of the epidural hematoma, the right pupil returned to 4 mm but was re-dilated to 5 mm after 10 min. The duration of cerebral herniation before the initiation of surgery was estimated to be approximately 150 min based on the first-aid records and surgical files. During the intraoperative evacuation of the hematoma, dural tension increased sharply, and the underneath brain tissue bulged out rapidly after the incision of the dura mater. Intraoperative CT showed a “flame”-shaped intracerebral hemorrhage in the involved brain tissue. After downregulating blood pressure, hyperventilation, and controlled cold compression onto the surgical area and the administration of large-dose steroids, the brain tissue was softened to some extent but remained herniated outside the craniotomy defect. Therefore, the herniated brain tissue had to be dissected. This patient scored 3 points on the Glasgow Outcome Scale at the 12-month follow-up and had left hemiplegia with decreased muscle strength (grade III).

### Case 2

**Figure 2:**
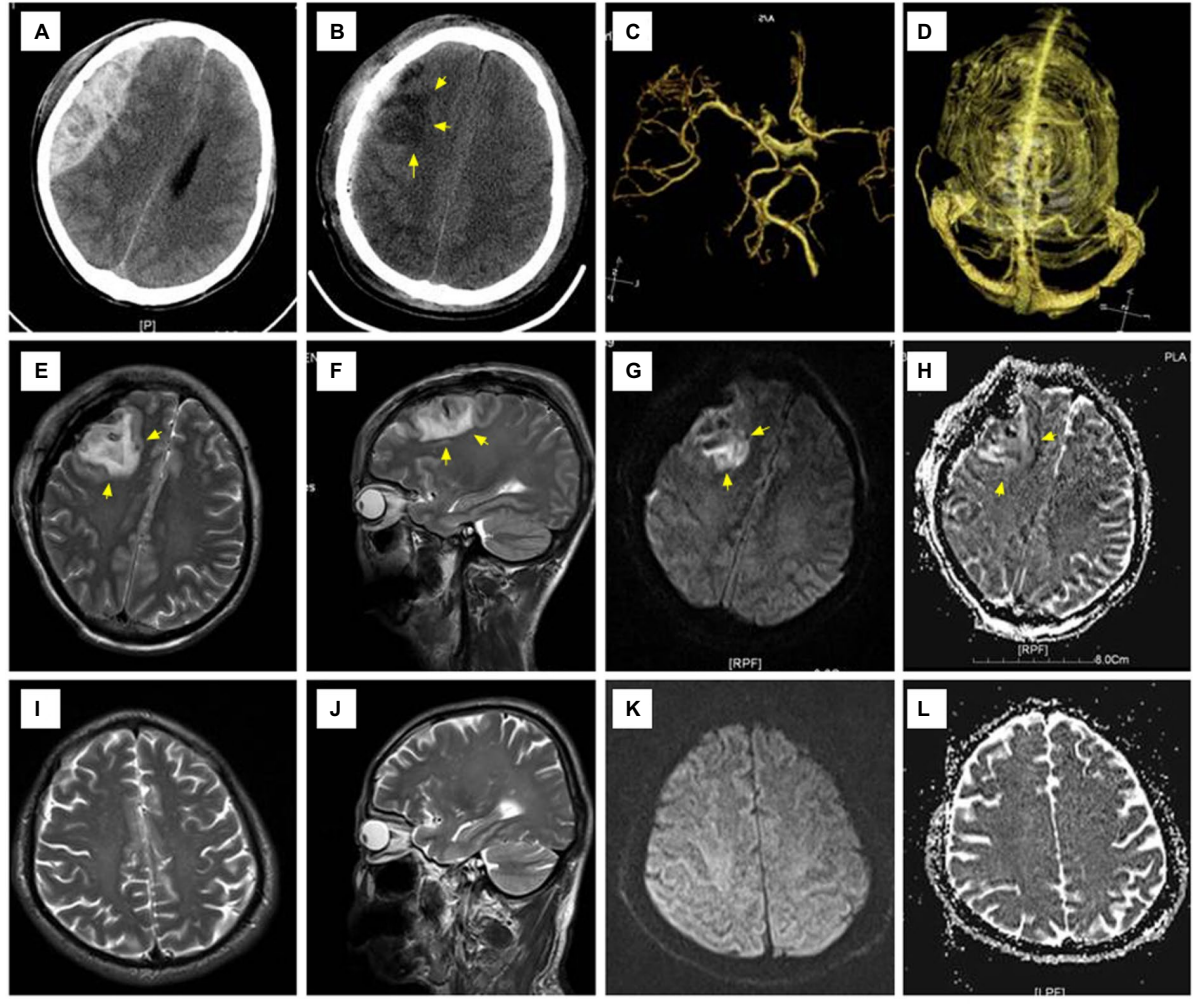
Representative radiographic images of Case 2. **(A)** An acute epidural hematoma occupying the right frontoparietal area; **(B)** CT examination performed 3 h after surgery showed patchy cerebral edema mixed with a few spotty areas of hemorrhage in the right frontal lobe; **(C,D)** CTA and CTV showed no obvious abnormalities; **(E–H)** T2WI showed a small amount of intracerebral hemorrhage and edema in the right frontal lobe with high signal on both DWI and ADC; **(I–L)** MRI re-examination 1 month after surgery showed that the lesion disappeared and the involved brain tissue recovered. CTA, CT angiography; CTV, CT venography; T1WI:T1 weighted imaging; DWI, diffusion-weighted imaging; ADC, apparent diffusion coefficient; MRI, magnetic resonance imaging.

A 35-year-old man fell from a high place and was admitted with an acute EDH in the right frontoparietal area with a volume of 118 ml and a thickness of 30 mm (Figure 2). Admission examination showed a 5-mm right pupil and a 3-mm left pupil. The patient scored seven points on the GCS scale, and the duration of herniation was estimated to be approximately 160 min. There was no preoperative shock. Preoperative burr hole drainage was performed. After surgical hematoma evacuation, the underlying dura mater showed a mild collapse; however, the tension was endurable, so the dura mater was not incised. CT examination performed 3 h later after surgery showed patchy cerebral edema mixing with a small amount of intraparenchymal hemorrhage at the compressed area. MRI examination showed signs of reperfusion injury in the right frontal lobe. Conservative treatment was effectual for the postoperative management of intracranial pressure, and no secondary surgery was required. This patient had a satisfactory recovery without any neurological dysfunction and scored five points on the GOS at the last follow-up 24 months later.

### Case 3

**Figure 3:**
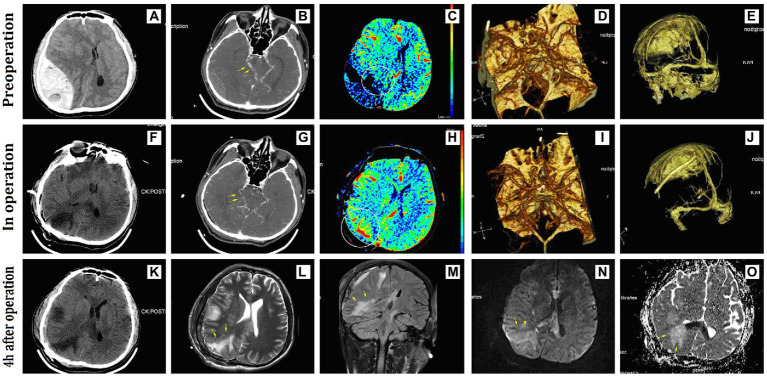
Representative radiographic images of Case 3. **(A)** A huge acute epidural hematoma occupied the right parietal-occipital area; **(B–D)** preoperative CTA showed compression and displacement of the right posterior cerebral artery; **(C)** preoperative CBF showed low perfusion due to hematoma compression in the right occipital lobe; **(E–J)** no significant compression on the venous sinus before and after surgery; **(F–K)** intra- and postoperative CT showed a low-density lesion in the right occipital lobe; **(G–I)** intra-operative CTA showed lessened displacement of the posterior cerebral artery; **(H)** intra-operative CBF showed increased perfusion in the right occipital lobe; **(L–M)** MRI examination performed 4 h after surgery showed long-signal shadows in the compressed areas of the right occipital lobe and corpus callosum on T2WI and FLAIR sequence; **(N,O)** the right occipital lobe showed long-signal on the DWI sequence and ADC sequence. CBF, cerebral blood flow; FLAIR, fluid-attenuated inversion recovery.

A 44-year-old man was admitted with an acute EDH in the right parietal-occipital area that was 144 ml in volume and 41 mm in thickness (Figure 3). Admission physical examination found that he had a 5-mm dilated right pupil and a 3-mm fixed-left pupil. This patient scored 4 points on the GCS scale, and the duration of herniation was estimated to be approximately 150 min before surgery. There was no preoperative shock. The dural pressure increased gradually after intra-operative hematoma evacuation. Intra-operative CT showed patchy areas of cerebral edema in the hematoma-compressed parenchyma and simultaneous CBF showed increased perfusion in the right occipital lobe. Intraoperative ICP management techniques were performed, including raising the head position, covering the focal dura mater with cold gauze, steroid administration, dehydration intervention, and hyperventilation. After those interventions, the tension of the dura mater stabilized, and, thus, it was not incised. This patient had a satisfactory recovery without an additional decompressive craniectomy and scored 4 points on the GOS scale at the last follow-up 18 months after discharge.

## Discussion

A patient who has an isolated EDH without severe intraparenchymal injury at the time of head injury usually recovers satisfactorily after timely and effective treatment (6). The largest prognostic threat comes from the development and progression of secondary brain injury induced predominantly by the mechanical compression of the epidural hematoma (7, 8), which calls for effectual pre-hospital first aid and a meticulous and well-organized surgical plan. In this study, we reported intracerebral hemorrhage or edema that occurs during surgical decompression or in the early post-surgical stage. We postulated that the underlying mechanism of this secondary brain injury might involve the impairment of cerebrovascular autoregulation of the decompensated intracranial vessels and the consequent focal hyperperfusion (9–12). The results showed that although the incidence of hyperperfusion injury is relatively low, it usually leads to unfavorable outcomes compared to those with normal perfusion.

The autoregulation function gets impaired during the early stage of a craniocerebral injury (13, 14), especially when CBF decreased markedly or hemodynamic disturbance occurred. As a result, dysfunction of cerebrovascular autoregulation becomes an important factor contributing to secondary brain injury in the early post-injury period (15). The mass effect of huge epidural hematoma directly compresses the underlying brain tissues and reduces blood perfusion to certain degrees, thereby producing a pathological basis of potential secondary brain injury. Because the ability of vasodilation of cerebral blood vessels is much greater than vasoconstriction (65 vs. 8–10% of the baseline caliber), it produces a greater influence on cerebral perfusion (16, 17). After hematoma evacuation, blood vessels in the locally compressed area are in an extreme state of tension restoration (10), which inevitably causes excessive brain blood reperfusion (18–20). Our study found that the secondary hemorrhage and edema in the brain parenchyma after surgical decompression were more pronounced in the area of direct compression, and their distribution was highly identical to the previous description.

Cerebral blood flow of intracranial vessels in the area directly compressed by hematoma was decreased markedly, where the local metabolic microenvironment underwent changes and progressively became worse with continued compression (21, 22). It indicated that the autoregulation function of the intracranial vessels involved is severely impaired, and the possibility of a fatal CBF burst following decompression and perfusion restoration is increasing gradually, which may even cause local congestive edema or hemorrhage. This justifies the rational supposition that the duration of brain herniation is a risk factor, as demonstrated in the current study. As longitudinal compression has the most pronounced impact on cortical branches, perforating branches of the intracranial vessels, and their communication (23, 24), it may involve higher-grade and more feeding arteries, eventually causing more pronounced and extensive injury to the autoregulatory function of the intracranial vessels. It is therefore theoretically reasonable to consider hematoma thickness as a risk factor for abnormal reperfusion injury. As the autoregulatory function of intracranial vessels in children and the elderly is either not well developed or decreased in compliance (25, 26), they are unable to make proper regulations to protect against abnormal hyperperfusion. In such patients who live, the course of the disease is usually short, the total volume of hematoma is relatively small, pressing time for vessels is transient, and the impairment to the autoregulation function of the blood vessels is relatively mild, therefore, reperfusion-induced secondary injury is also relatively mild.

Compared with the aforementioned uncontrollable influencing factors, we first notice that the amplitude of instant change in CBF reperfusion is controllable to some extent. It is also an important factor affecting CBF reperfusion in patients with abnormal cerebral vascular autoregulation (27–29). Tamaki et al. (30) found that the quick evacuation of the hematoma could decrease the intracranial pressure sharply and, at the same time, trigger a severe aggravation of hemodynamic change, thus worsening the overload injury to the decompensated cerebral vessels. Based on this understanding, they advanced the concept of the gradual evacuation of intracranial hematoma to avoid instant vigorous fluctuations of intracranial pressure and CBF reperfusion. To the best of our knowledge, we are the first to point out pathological mechanisms of vigorous change in CBF reperfusion during the process of EDH evacuation. Based on this finding, we also propose a therapeutic strategy to control the amplitude of CBF during instant cerebral blood reperfusion, which is expected to reduce the occurrence of abnormal reperfusion injury. For this reason, we make use of the core idea of controlled decompression and have made some beneficial explorations and practices in our neurological center with satisfactory clinical outcomes (31). First, we conducted a preliminary skull drilling to suction a portion of the EDH for moderate decompression before the formal craniotomy when managing patients with cerebral herniation from a huge epidural hematoma. The process can not only release fatal compression on the brain stem quickly and shorten the duration of cerebral herniation but also ensure stable gradient release of intracranial pressure by controlling the amount and rate of hematoma drainage. It is also useful to control the amplitude of instant change of CBF reperfusion in the decompressed area and attenuate the second strike on the local cerebral vessels in which autoregulatory ability has been impaired, thus creating an opportunity to prevent abnormal reperfusion injury following the decompression procedures. Second, to assess the cause of increased dural tension during decompression and especially differentiate it from intracranial hypertension due to distal hemorrhage, subdural hematoma formation, and venous reflux dysfunction to avoid the disastrous consequence from decompression by imprudent incision of the dura mater, we used the mobile CT scanner (NeuroLogica, USA) for intra-operative examination to exclude the latter causes and then raised the head position during surgery, covered the local dura with ice saline, and employed steroids, dehydration, and hyperventilation without opening the dura. Such patients often achieved satisfactory therapeutic outcomes with these techniques. In high-risk patients who are preoperatively assessed as having abnormal reperfusion injury, timely postoperative CT, CTP, or even MRI scanning re-examinations are often necessary. Even in patients who need a second craniotomy, surgical time should be assessed correctly. Above all, abnormal reperfusion injury during EDH evacuation can be minimized by positive pretreatment, which will eventually improve the clinical outcome.

Our study has several limitations that should be accounted for. Due to the retrospective nature of the study, conclusions related to risk factors for prediction should be taken with caution. Furthermore, allowing for rarity, the sample was slightly inadequate, and several patients were lost to follow-up adding to the weaknesses of the analysis. Consequently, an insufficiently detailed statistical analysis was conducted to interpret the possible factors that appeared to influence the prediction.

In summary, abnormal reperfusion injury following EDH evacuation is a common surgical complication that plays an extremely important role in restricting the surgical outcome. A further study in exploring the underlying pathological mechanism and contributing factors is crucial for perfecting the surgical plan and improving the surgical outcome.

## Data availability statement

The raw data supporting the conclusions of this article will be made available by the authors, without undue reservation.

## Ethics statement

Ethical review and approval was not required for the study on human participants in accordance with the local legislation and institutional requirements. The patients/participants provided their written informed consent to participate in this study.

## Author contributions

WH, W-hW, and JL contributed to the conception and design of the study. YZ organized the database. FL performed the statistical analysis. WH wrote the first draft of the manuscript. W-hW, FL, L-SH, and J-ML wrote sections of the manuscript. All authors contributed to the article and approved the submitted version.

## Funding

This study was supported by the Natural Science Foundation of Fujian Province, China (2018 J01152).

## Conflict of interest

The authors declare that the research was conducted in the absence of any commercial or financial relationships that could be construed as a potential conflict of interest.

## Publisher’s note

All claims expressed in this article are solely those of the authors and do not necessarily represent those of their affiliated organizations, or those of the publisher, the editors and the reviewers. Any product that may be evaluated in this article, or claim that may be made by its manufacturer, is not guaranteed or endorsed by the publisher.

## References

[1] Yang C Hui J Xie L Feng J Jiang J. Comparative effectiveness of different surgical procedures for traumatic acute epidural haematoma: study protocol for prospective, observational real-world treatments of AEDH in large-scale surgical cases (PORTALS-AEDH). BMJ Open. (2022) 12:e051247. doi: 10.1136/bmjopen-2021-051247, PMID: 35264341 PMC8915281

[2] Maugeri R Anderson DG Graziano F Meccio F Visocchi M Iacopino DG. Conservative vs. surgical Management of Post-Traumatic Epidural Hematoma: a case and review of literature. Am J Case Rep. (2015) 16:811–7. doi: 10.12659/AJCR.895231, PMID: 26567227 PMC4652627

[3] Wang WH Hu LS Lin H Li J Luo F Huang W. Risk factors for post-traumatic massive cerebral infarction secondary to space-occupying epidural hematoma. J Neurotrauma. (2014) 31:1444–50. doi: 10.1089/neu.2013.3142, PMID: 24773559

[4] Zhang S Wang S Wan X Liu S Shu K Lei T. Clinical evaluation of post-operative cerebral infarction in traumatic epidural haematoma. Brain Inj. (2017) 31:215–20. doi: 10.1080/02699052.2016.1227088, PMID: 28055227

[5] Otani N Takasato Y Masaoka H Hayakawa T Yoshino Y Yatsushige H. Surgical outcome following a decompressive craniectomy for acute epidural hematoma patients presenting with associated massive brain swelling. Acta Neurochir Suppl. (2010) 106:261–4. doi: 10.1007/978-3-211-98811-4_49, PMID: 19812961

[6] Bricolo AP Pasut LM. Extradural hematoma: toward zero mortality. A prospective study. Neurosurgery. (1984) 14:8–12. doi: 10.1227/00006123-198401000-00003, PMID: 6694798

[7] Ciurea AV Tascu A Brehar FM Nuteanu L Rizea R. A life threatening problem in infants: supratentorial epidural hematoma. J Med Life. (2009) 2:191–5. 20108539 PMC3018979

[8] Xiao B Ma MY Duan ZX Liu JG Chen RP Mao Q. Could a traumatic epidural hematoma on early computed tomography tell us about its future development? A multi-center retrospective study in China. J Neurotrauma. (2015) 32:487–94. doi: 10.1089/neu.2013.3297, PMID: 25050450

[9] Gross PM Heistad DD Strait MR Marcus ML Brody MJ. Cerebral vascular responses to physiological stimulation of sympathetic pathways in cats. Circ Res. (1979) 44:288–94. doi: 10.1161/01.RES.44.2.288, PMID: 761309

[10] Osol G Halpern W. Myogenic properties of cerebral blood vessels from normotensive and hypertensive rats. Am J Phys. (1985) 249:H914–21. doi: 10.1152/ajpheart.1985.249.5.H914, PMID: 4061668

[11] Pannier JL Weyne J Leusen I. Effects of changes in acid-base composition in the cerebral ventricles on local and general cerebral blood flow. Eur Neurol. (1971) 6:123–6. doi: 10.1159/000114479, PMID: 5153414

[12] Claassen J Thijssen D Panerai RB Faraci FM. Regulation of cerebral blood flow in humans: physiology and clinical implications of autoregulation. Physiol Rev. (2021) 101:1487–559. doi: 10.1152/physrev.00022.2020, PMID: 33769101 PMC8576366

[13] Hlatky R Valadka AB Robertson CS. Intracranial pressure response to induced hypertension: role of dynamic pressure autoregulation. Neurosurgery. (2005) 57:917–23. doi: 10.1227/01.NEU.0000180025.43747.fc, PMID: 16284561

[14] Trofimova S Trofimov A Dubrovin A Agarkova D Trofimova K Dobrzeniecki M. Assessment of cerebral autoregulation in the Perifocal zone of a chronic subdural hematoma. Acta Neurochir Suppl. (2021) 131:51–4. doi: 10.1007/978-3-030-59436-7_11, PMID: 33839817 PMC8086812

[15] Zipfel J Hockel KL Gerbig I Heimberg E Schuhmann MU Neunhoeffer F. Impaired autoregulation following resuscitation correlates with outcome in pediatric patients: a pilot study. Acta Neurochir Suppl. (2021) 131:97–101. doi: 10.1007/978-3-030-59436-7_21, PMID: 33839827

[16] Preiksaitis A Krakauskaite S Petkus V Rocka S Chomskis R Dagi TF. Association of Severe Traumatic Brain Injury Patient Outcomes with Duration of cerebrovascular autoregulation impairment events. Neurosurgery. (2016) 79:75–82. doi: 10.1227/NEU.0000000000001192, PMID: 26695090

[17] Rangel-Castilla L Gasco J Nauta HJ Okonkwo DO Robertson CS. Cerebral pressure autoregulation in traumatic brain injury. Neurosurg Focus. (2008) 25:E7. doi: 10.3171/FOC.2008.25.10.E7 18828705

[18] Crippa IA Creteur J Smielewski P Taccone FS Czosnyka M. Delay of cerebral autoregulation in traumatic brain injury patients. Clin Neurol Neurosurg. (2021) 202:106478. doi: 10.1016/j.clineuro.2021.106478, PMID: 33454499

[19] Svedung Wettervik T Howells T Hillered L Rostami E Lewén A Enblad P. Autoregulatory or fixed cerebral perfusion pressure targets in traumatic brain injury: determining which is better in an energy metabolic perspective. J Neurotrauma. (2021) 38:1969–78. doi: 10.1089/neu.2020.7290 33504257

[20] Schmidt EA Czosnyka M Steiner LA Balestreri M Smielewski P Piechnik SK. Asymmetry of pressure autoregulation after traumatic brain injury. J Neurosurg. (2003) 99:991–8. doi: 10.3171/jns.2003.99.6.0991, PMID: 14705726

[21] Robertson CS Goodman JC Grossman RG. Blood flow and metabolic therapy in CNS injury. J Neurotrauma. (1992) 9:S579–94. 1613815

[22] Sharma HS Muresanu DF Lafuente JV Nozari A Patnaik R Skaper SD. Pathophysiology of blood-brain barrier in brain injury in cold and hot environments: novel drug targets for neuroprotection. CNS Neurol Disord Drug Targets. (2016) 15:1045–71. doi: 10.2174/1871527315666160902145145, PMID: 27592625

[23] Djulejić V Marinković S Milić V Georgievski B Rašić M Aksić M. Common features of the cerebral perforating arteries and their clinical significance. Acta Neurochir. (2015) 157:1393. doi: 10.1007/s00701-015-2462-0, PMID: 26066534

[24] Liebeskind DS Caplan LR. Intracranial arteries—anatomy and collaterals. Front Neurol Neurosci. (2016) 40:1–20. doi: 10.1159/000448264 27960167

[25] Melamed E Lavy S Bentin S Cooper G Rinot Y. Reduction in regional cerebral blood flow during normal aging in man. Stroke. (1980) 11:31–5. doi: 10.1161/01.STR.11.1.31, PMID: 7355426

[26] Petkus V Preiksaitis A Chaleckas E Chomskis R Zubaviciute E Vosylius S. Optimal cerebral perfusion pressure: targeted treatment for severe traumatic brain injury. J Neurotrauma. (2020) 37:389–96. doi: 10.1089/neu.2019.6551, PMID: 31583962

[27] Kasprowicz M Czosnyka M Poplawska K Reinhard M. Cerebral arterial time constant recorded from the MCA and PICA in Normal subjects. Acta Neurochir Suppl. (2016) 122:211–4. doi: 10.1007/978-3-319-22533-3_42, PMID: 27165908

[28] Zeiler FA Aries M Czosnyka M Smielewski P. Cerebral autoregulation monitoring in traumatic brain injury: an overview of recent advances in personalized medicine. J Neurotrauma. (2022) 39:1477–94. doi: 10.1089/neu.2022.0217, PMID: 35793108

[29] Small C Lucke-Wold B Patel C Abou-Al-Shaar H Moor R Mehkri Y. What are we measuring? A refined look at the process of disrupted autoregulation and the limitations of cerebral perfusion pressure in preventing secondary injury after traumatic brain injury. Clin Neurol Neurosurg. (2022) 221:107389. doi: 10.1016/j.clineuro.2022.107389, PMID: 35961231

[30] Tamaki T Node Y Yamamoto Y Teramoto A. Cardiopulmonary hemodynamic changes during acute subdural hematoma evacuation. Neurol Med Chir (Tokyo). (2006) 46:219–25. doi: 10.2176/nmc.46.219, PMID: 16723813

[31] Chen T Qian X Zhu J Yang LK Wang YH. Controlled decompression attenuates compressive injury following traumatic brain injury via TREK-1-mediated inhibition of necroptosis and Neuroinflammation. Oxidative Med Cell Longev. (2021) 2021:4280951–17. doi: 10.1155/2021/4280951, PMID: 34790287 PMC8592713

